# Genotypic analysis of two hypervariable human cytomegalovirus genes

**DOI:** 10.1002/jmv.21241

**Published:** 2008-09

**Authors:** Amanda J Bradley, Ida J Kovács, Derek Gatherer, Derrick J Dargan, Khaled R Alkharsah, Paul KS Chan, William F Carman, Martin Dedicoat, Vincent C Emery, Colin C Geddes, Giuseppe Gerna, Bassam Ben-Ismaeil, Steve Kaye, Alistair McGregor, Paul A Moss, Rozalia Pusztai, William D Rawlinson, Gillian M Scott, Gavin WG Wilkinson, Thomas F Schulz, Andrew J Davison

**Affiliations:** 1 MRC Virology Unit, Institute of Virology, University of GlasgowGlasgow, United Kingdom; 2 Department of Medical Microbiology and Immunobiology, University of SzegedSzeged, Hungary; 3 Institut für Virologie, Medizinische Hochschule HannoverHannover, Germany; 4 Department of Microbiology, The Chinese University of Hong Kong, Prince of Wales Hospital, Shatin, New Territories, Hong Kong Special Administrative RegionChina; 5 West of Scotland Specialist Virology Centre, Gartnavel General HospitalGlasgow, United Kingdom; 6 Ngwelezane Hospital, EmpangeniKwaZulu/Natal, South Africa; 7 Centre for Virology, Division of Infection and Immunity, Royal Free and University College Medical School, HampsteadLondon, UK; 8 Renal Unit, Western InfirmaryGlasgow, United Kingdom; 9 Servizio di Virologia, Fondazione IRCCS Policlinico San MatteoPavia, Italy; 10 Viral Disease Programme, Medical Research Council Laboratories, Atlantic BoulevardBanjul, The Gambia; 11 Division of Infectious Diseases, University of Minnesota Department of Pediatrics, Center for Infectious Diseases and Microbiology Translational Research, McGuire Translational Research FacilityMinneapolis, Minnesota; 12 Cancer Research UK Institute for Cancer Studies, The University of Birmingham, EdgbastonBirmingham, United Kingdom; 13 Virology Division, Department of Microbiology, Prince of Wales Hospital, RandwickNew South Wales, Australia; 14 Department of Medical Microbiology, Wales School of Medicine, Cardiff UniversityHeath Park, Cardiff, United Kingdom

**Keywords:** herpesvirus, variation, genotype, chemokine, glycoprotein

## Abstract

Most human cytomegalovirus (HCMV) genes are highly conserved in sequence among strains, but some exhibit a substantial degree of variation. Two of these genes are UL146, which encodes a CXC chemokine, and UL139, which is predicted to encode a membrane glycoprotein. The sequences of these genes were determined from a collection of 184 HCMV samples obtained from Africa, Australia, Asia, Europe, and North America. UL146 is hypervariable throughout, whereas variation in UL139 is concentrated in a sequence encoding a potentially highly glycosylated region. The UL146 sequences fell into 14 genotypes, as did all previously reported sequences. The UL139 sequences grouped into 8 genotypes, and all previously reported sequences fell into a subset of these. There were minor differences among continents in genotypic frequencies for UL146 and UL139, but no clear geographical separation, and identical nucleotide sequences were represented among communities distant from each other. The frequent detection of multiple genotypes indicated that mixed infections are common. For both genes, the degree of divergence was sufficient to preclude reliable sequence alignments between genotypes in the most variable regions, and the mode of evolution involved in generating the genotypes could not be discerned. Within genotypes, constraint appears to have been the predominant mode, and positive selection was detected marginally at best. No evidence was found for linkage disequilibrium. The emerging scenario is that the HCMV genotypes developed in early human populations (or even earlier), becoming established via founder or bottleneck effects, and have spread, recombined and mixed worldwide in more recent times.

## Introduction

Human cytomegalovirus (HCMV; family *Herpesviridae*, subfamily *Betaherpesvirinae*, genus *Cytomegalovirus*, species *Human herpesvirus* 5) is ubiquitous and host–specific. Infection is asymptomatic for most people, but can result in serious disease in immunocompromised individuals and congenitally infected newborns. The minimally passaged Merlin strain is considered best to represent wild–type HCMV, and has a 236 kbp genome that is predicted to contain approximately 165 protein–encoding genes [Dolan et al., 2004].

Most genes are highly conserved in sequence between HCMV strains, but a number of genes predicted to encode membrane-associated or secreted proteins are characterized by a striking degree of variability, as revealed by examination of individual genes [reviewed in Pignatelli *et al*., 2004] and by whole genome comparisons [ Murphy et al., 2003; Dolan et al, 2004]. Various studies have attempted to make connections between the genotypes of hypervariable genes and disease outcome, and overall the conclusions reached are unclear or contradictory [reviewed in Puchhammer-Stöckl and Görzer, 2006]. The apparently random association of genotypes at different loci (that is, the absence of linkage disequilibrium), presumably as a result of recombination during HCMV evolution, limits any conclusions to the specific gene under study, except in some cases where genes are very close to each other or encode interacting hypervariable proteins [Rasmussen *et al*., 2002, 2003]. A further complicating factor is the occurrence of multiple HCMV genotypes in individuals, including immunocompromised patients, pregnant women and congenitally infected newborns [for recent papers, see Rasmussen et al., 2003; Hassan-Walker et al., 2004; Stanton et al., 2005; Puchhammer-Stöckl et al., 2006; Iwasenko et al., 2007].

One of the most variable HCMV genes is UL146, which encodes a chemokine designated vCXC-1. This gene is variable throughout its length [Penfold et al., 1999; Prichard et al., 2001; Hassan-Walker et al., 2004; Arav-Boger et al., 2005, 2006; Stanton et al., 2005; He et al., 2006; Lurain et al., 2006], and 14 genotypes have been catalogued [Dolan et al., 2004]. In strain Toledo, UL146 encodes a functional chemokine that is capable of neutrophil degranulation, chemotaxis and calcium mobilization. This protein contains an ELRCXC motif, which has been shown to be essential for receptor binding and IL-8 activity [Clark-Lewis et al., 1991]. vCXC-1 binds to human CXCR2 and is comparable in its activities to CXC chemokines IL-8 and gro-α [Penfold et al., 1999]. The function of vCXC-1 may be to facilitate dissemination of the virus through its ability to attract monocytes to the initial site of infection. Thus, the virus could undermine the effectiveness of antiviral immunity by manipulating the host chemokine system and, together with other virus–encoded molecular mimics, suppressing the immune system.

The most variable gene in the vicinity of UL146 is UL139, which is located 5.2 kbp distant and is predicted to encode a type I membrane glycoprotein. Variability is concentrated in a region of the ectodomain [Dolan et al., 2004]. A recent study of 26 HCMV strains isolated in China described three major genotypes, with two of these divided into subtypes [Qi et al., 2006].

The aims of the present study were to investigate whether additional UL146 genotypes exist in a large number of clinical samples obtained from a wide range of locations and clinical settings, and to define the range of UL139 genotypes in these samples. Ancillary interests were to examine the relative frequencies and geographical distribution of genotypes, to assess whether infections with more than one HCMV strain are common, and to investigate the evolution of UL146 and UL139.

## MATERIALS AND METHODS

### Virus DNA Collection

A collection of 184DNAsamples was derived from 179 anonymized clinical samples obtained in various geographical locations in accordance with local ethical guidelines, plus 5 commonly used laboratory strains (Davis, Merlin, TB40/E, Toledo and Towne). Details of the 171 samples in the collection that yielded sequence data are available on request, and include the age, sex, and pathology of the patient, the clinical source of the sample, and the UL146 and UL139 genotypes determined. The samples numbered 18 from Australia, 10 from Hong Kong, 6 from Germany, 13 from England, 18 from The Gambia, 24 from Hungary, 7 from Italy, 6 from The Netherlands, 41 from Scotland, 5 from the USA, 8 from Wales, and 15 from South Africa. A minority of strains (40) had been passaged in human fibroblast cell culture, either as routine diagnostic specimens or as laboratory strains. DNA was extracted by standard methods from body tissues, urine, saliva or infected cells. The South African samples were obtained from the saliva of mothers (10 of whom tested HIV-negative) attending rural clinics in KwaZulu/Natal [Dedicoat et al., 2004]. Since these were available in very limited amounts and contained low numbers of HCMV genomes, whole genome amplification using a REPLIg kit (Qiagen, Crawley, UK)was carried out prior to PCR amplification.

### PCR Amplification

UL146 and UL139 were amplified separately by single round or nested PCR, using primers in conserved regions (Table 1). Single (and first) round PCR of UL146 using AB4 and A162 generated a product of approximately 1 kbp, and second round PCR using UL146–4A and UL146–3A yielded an 800 bp product. Single (and first) round PCR of UL139 using AB1 and AB2generated an 800 bp product, and nested PCR using UL140–3A and UL140–11A yielded a 500 bp product. UL140–11A is located within the UL139 coding region, and as a consequence the sequences obtained using nested PCR (approximately 40% of the total) lacked 29 amino acid–encoding codons from the highly conserved C terminus.

**TABLE I:** Primers Used for PCR and Sequencing

Gene	Primer	Sequence(5′–3′)	Genome location^a^
UL146	AB4	TAGACACTACGTCGTAAATG	180494–180513
UL146	A162	TGTAGAATTAGTCTAGATTCCTGA	181524–181501
UL146	UL146–4A	GCTTGCGCGTTAGGATTGAGACAC	180571–180594
UL146	UL146–3A	ATACCGGATATTACGAATT	181341–181323
UL139	AB1	GTCATTGTGAAAGTGACGTCTCAG	186389–186412
UL139	AB2	ATCTACTGTAAACCCTCTGCTCTG	187148–187125
UL139	UL140–11A	GCGGCATTGGTGTACGCGTG	186553–186572
UL139	UL140–3A	GTGGAAATTTTTACGTCATT	187077–187058

a With reference to RefSeq accession NC_006273.2 (HCMV strain Merlin).

For the single (and first) round, 1µl ofDNAwas added to the PCR reaction mixture, which consisted of 40µl of water, 5µl of buffer, 1µl of10µM dNTPs, 1µl of each the two primers (10µM) and 1µl (1U) of DNA polymerase (Advantage 2, BD Clontech, Basingstoke, UK). The conditions for amplification were 95°Cfor 2 min followed by 35 cycles of 95°Cfor 2 min, 60°Cfor 30 sec and 68°Cfor 1 min. Second round PCR utilized 1µl of first round PCR products as template amplified under the same conditions. PCR reactions were set up in a dedicated, PCR product–free room. Approximately one–third of the samples were tested on three separate occasions to assess reproducibility.

### Purification, Cloning, and Sequencing of PCR Products

PCR products were separated by agarose gel electrophoresis. Appropriate DNA fragments were excised, purified using a Geneclean turbo kit (Q Biogene, Cambridge, UK), and eluted using 100µl of nucleasefree water. The single round or second round primers were used for direct sequencing.

In some cases, including those where direct sequencing indicated the presence of more than one genotype of UL146 or UL139, fragments were cloned using a pGEM–T kit (Promega, Southampton, UK). Following ligation and transformation into chemically competent *E. coli* TOP 10 cells, 5 recombinant colonies were picked and grown overnight at 37°C in 2YT-broth containing 100°g/ml ampicillin. Plasmid DNA was purified using a QIAprep Spin miniprep kit (Qiagen). Plasmid inserts were sequenced using universal forward and reverse primers. Sequencing was carried out on both DNA strands using a BigDye terminator kit (Applied Biosystems, Warrington, UK) in an ABI 3730 instrument. Samples containing multiple strains were identified by the derivation of plasmids representing different genotypes of UL146 or UL139.

### Sequence Analysis

Sequence chromatograms were viewed using Editview (Applied Biosystems) and analyzed using Pregap4 and Gap4 [Staden et al., 2000]. Nucleotide and imputed amino acid sequences were aligned using CLUSTAL W [Thompson et al., 1994] and MAFFT[Katoh et al., 2005]. Full–length sequences were used for the UL146 data and a subset of the UL139 data, and another subset of the UL139 data was analyzed using sequences lacking the conserved C terminus. MEGA4.0 [Tamura et al., 2007] was used for the generation of phylogenetic trees. Frequencies of nonsynonymous and synonymous differences per site (dN and dS, respectively) and degree of sequence variability (nucleotide and amino acid) were investigated using Swaap 1.0.1 [Pride, 2004], DnaSP 4.10.9 [Rozas et al., 2003], and MEGA4.0. dN/dS ratios and probabilities of positive selection were assessed using PAML 3.15 [Yang, 1997]. Signal peptide and transmembrane sequences were predicted using Phobius [Kall et al., 2004]

### Statistical Anslysis

Sample origin was divided into four regions (Africa, Asia, Europe, and Australia) for assessment of the geographical distribution of genotypes. Chi-square tests were used to assess the significance of variability of genotype frequencies among regions. Yates' correction for continuity was applied to chi–square tests in cases where the expected values fell below 5. Similarly, Chisquare tests with Yates' correction were applied to 60 samples where single genotypes were detected for both UL146 and UL139, in order to test for linkage disequilibrium. Samples containing mixed infections were excluded from this analysis.

## RESULT

### UL146 and UL139 Sequences

The UL146 and UL139 genotypes in 184 samples were investigated by PCR and sequencing using primers in conserved regions. UL146 was amplified from 159 samples and sequences were determined from 134, and UL139 was amplified from 168 samples and sequences determined from 131.Atotal of 13 samples failed to yield products from either gene. Since some samples contained more than one virus strain, totals of 182 UL146 sequences and 183 UL139 sequences were obtained. Alignment and phylogenetic analyses involved the 350 UL146 sequences and 300 UL139 sequences derived from the present study or reported by others in the literature [Cha et al., 1996; Davison et al., 2003; Dolan et al., 2004; Arav-Boger et al., 2005, 2006; Stanton et al., 2005; He et al., 2006; Lurain et al., 2006; Qi et al., 2006] or public sequence databases [AY999242-AY999271, AY805250-AY805303, AY818250-AY818255, Mao et al.; DQ229942–DQ229948, Ruan et al.; DQ180366, DQ180358, DQ180374, DQ180386, Zhou et al.].

### UL146 Genotypes

The UL146 coding sequences range in length from 114 to 126 codons, and phylogenetic analyses indicated that all fall into the 14 genotypes defined previously and designated G1–G14 [Dolan et al., 2004]. Amino acid sequence variation among genotypes is high (p=0.521, where p is protein diversity from MEGA4.0), whereas within each genotype it is low (p=0–0.051 with a mean of 0.017)(Table II).

**TABLE II:** UL146 Diversity

			Alignment lenth^a^		Diversity		
					
Genotype	Samples	Frequences(%)	DNA	Protein	DNA^b^	Protein^c^	dN/dS^d^
G1	34	9.71	345	115	0.011	0.026	1.19^e^
G2	25	7.14	360	120	0.002	0.005	1.48
G3	10	2.86	375	125	0.010	0.016	0.50
G4	8	2.29	369	123	0.004	0.006	0.26
G5	16	4.57	348	116	0.007	0.012	0.71
G6	2	0.57	351	117	0.029	0.051	ND
G7	57	16.3	354	118	0.011	0.015	1.29^e^
G8	22	6.29	342	114	0.006	0.008	0.38
G9	49	14	351	117	0.017	0.032	0.94
G10	12	3.43	291	97	0.003	0.005	0.30
G11	19	5.43	339	113	0.007	0.015	0.50
G12	43	12.3	354	118	0.016	0.018	0.45
G13	47	13.4	357	119	0.007	0.015	1.58
G14	6	1.71	354	118	0	0	ND
All	350	100	225	75	0.642	0.521	0.27^f^

a Gaps removed

b Jukes–Cantor *Pi* from DnaSP 4.10.9

c Protein diversity p from MEGA4.0

d dN/dS(omega) from PAML 3.15 under the single–rate model.ND, not determined

e Five percent significance for positive selection

f Calculated from a comparison of a single of a member of each genotype

Differences in overall genotypic frequencies were observed (Table II). For example, G7 was detected in 16% of samples and G6 in fewer than 1%.

### UL139 Genotypes

**Figure 1:**
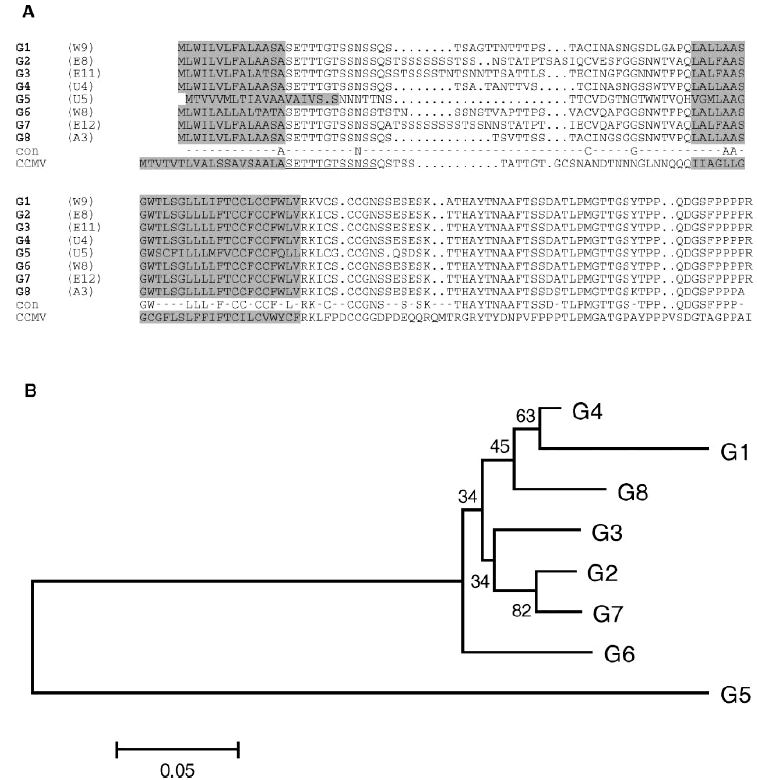
Phylogenetic analysis of UL139.A:Alignment (CLUSTAL W) of amino acid sequences representing the eight genotypes. Predicted signal peptide and transmembrane sequences are highlighted in gray. Completely conserved residues are indicated in the consensus row (con). Below this is the CCMV sequence, which is included to illustrate conservation of the SETTTGTSSNSS motif (underlined). The CCMV sequence [Davison et al., 2003] provided is theC–terminal portion (final 12 residues not shown) of a larger protein, the N–terminal portion of which lacks a counterpart in HCMV but is related to a protein (encoded by gene rh174) in rhesus cytomegalovirus. B: Unrooted neighborjoining tree for the HCMV amino acid sequences shown in (A), computed using Mega4.0 (Poisson correction method with gaps removed). Bootstrap values (out of 100) are shown, and values below 70 indicate regions of unresolved branching order. The scale bar indicates the number of amino acid substitutions per site.

The UL139 coding sequences range in length from 124 to 148 codons, and phylogenetic analyses indicated that all fall into 8 genotypes designated G1–G8. Fig. 1A shows a predicted amino acid sequence alignment of the primary translation products of one representative of each genotype. Figure B shows a phylogenetic tree constructed using these sequences.

The protein encoded by each HCMV UL139 genotype contains a putative signal peptide sequence and a transmembrane region. Variation is concentrated in the N–terminal portion of the protein. Amino acid sequence variation between genotypes is high (p=0.275), whereas within each genotype it is low (p=0.007–0.095 with a mean of 0.025) (Table3). Variation within genotypes tends to be higher in UL139 than in UL146, but that among genotypes is lower. Sequences in G1 exhibit a greater level of variation than those in the other genotypes.

**TABLE III:** UL139 Diversity

			Alignment lenth^a^		Diversity		
					
Genotype	Samples	Frequency(%)	DNA	Protein	DNA^b^	Protein^c^	dN/dS^d^
G1	48	16	255	85	0.059	0.095	0.82^e^
G2	82	27.33	240	80	0.009	0.009	0.76
G3	29	9.66	339	113	0.014	0.013	0.34
G4	68	22.66	201	67	0.023	0.015	0.76
G5	28	9.33	255	124	0.018	0.024	0.65
G6	24	8	312	104	0.010	0.013	1.08
G7	14	4.66	237	79	0.006	0.015	2.38
G8	7	2.33	228	140	0.007	0.007	0.19
All	300	100	153	51	0.285	0.275	0.48^f^

a Gaps removed

b Jukes–Cantor *Pi* from DnaSP 4.10.9

c Protein diversity p from MEGA4.0.

d dN/dS(omega) from PAML 3.15 under the single–rate model

e One persent significance for positive selection

f Calculated from a comparision of a single member of each genotype

Differences in overall genotypic frequencies were observed (Table III). For example, G2 was detected in 27% of samples and G8 in fewer than 3%.

### Assessment of Positive Selection

In order to assess positive selection (i.e., for amino acid sequence diversity), the dN/dS ratio was calculated for each UL146 and UL139 genotype (Tables II and III). Positive selection was detected at the 1% significance level only in UL139 G1, and at the 5% level in UL146 G7 and G1. UL139 G6 and G7 and UL146 G2 and G13 also had values of dN/dS>1, but these were not statistically significant. Only in UL139G1was an amino acid residue identified as under positive selection, although this was at position 12 in the predicted signal peptide sequence. Thus, evidence for positive selection is marginal, and it seems unlikely that this mode of diversification has featured in the evolution of UL139 and UL146 since the genotypes arose. No strong evidence emerged for positive selection in formal comparisons among genotypes (i.e., as a factor in emergence of the genotypes), but it must be registered that variation was so large as to confound reliable sequence alignments.

### Geographical Distribution of Genotypes

The sequence data derived in the present work were divided into four groups representing strains obtained from Africa, Asia, Australia, and Europe. Insufficient sample numbers were obtained from America to war–rant inclusion. Observation of frequencies initially suggested no significant differences in the distribution of UL146 and UL139 genotypes among continents (Tables IV and V). However, this conclusion may have been compromised by insufficient sample numbers from some areas (particularly Asia) and lack of information on the ethnic origin of samples. This shortcoming is highlighted by the observation that regions (specifically Europe) for which a larger sample size was tested displayed greater genotypic diversity. Nonetheless, UL146G13 appears somewhat more common in African samples than in European samples (11 out of 45 sequences were detected in the former and 17 out of 104 in the latter), UL146 G10 and G11 appear to be restricted to Europe (8 and 3 samples, respectively), and the single sample of UL146 G6 originated from Asia.

**TABLE IV:** Geographical Distribution of UL146 Genotypes

Genotype	Africa	Asia	Europe	Australia
G1	4	2	7	0
G2	2	1	7	2
G3	4	0	2	0
G4	1	0	4	2
G5	3	0	2	1
G6	0	1	0	0
G7	5	6	21	1
G8	2	0	5	0
G9	6	2	11	3
G10	0	0	8	0
G11	0	0	3	0
G12	5	1	16	0
G13	11	0	17	0
G14	2	0	1	0
Total=177	45	13	104	15

**TABLE V:** Geographical Distribution of UL139 Genotypes

Genotype	Africa	Asia	Europe	Australia
G1	8	2	16	2
G2	9	1	23	5
G3	1	4	7	0
G4	8	5	23	5
G5	10	3	14	0
G6	0	1	11	1
G7	3	0	5	5
G8	2	2	1	1
Totals=178	41	18	100	19

Identical nucleotide sequences were frequently obtained from geographically distant and presumably epidemiologically unrelated patients. For example, certain samples from The Gambia, Scotland, and Hungary contained identical UL146 G12 sequences. Also, UL139 G2, which was identified in 27% of samples, was represented by identical sequences from Hungary, The UK, and The Gambia.

### Linkage Disequilibrium Between UL146 and UL139

Potential linkage disequilibrium was investigated in 60 strains for which single genotypes of both UL146 and UL139 were obtained. Of 112 possible genotype pairs, 41 were observed at least once (Table VI). No statistical significance was obtained for the observed distribution of genotype pairs versus a null hypothesis of independent assortment, indicating an absence of linkage disequilibrium.

**TABLE VI:** Analysis of Linkage Disequilibrium

	UL139 genotype							
	
UL146 genotype	G1	G2	G3	G4	G5	G6	G7	G8
G1	1	0	0	3	1	0	0	0
G2	1	1	0	0	0	0	0	0
G3	1	0	0	0	0	0	0	0
G4	0	0	1	1	0	0	0	0
G5	0	1	0	1	0	0	1	0
G6	0	0	0	0	0	0	0	0
G7	1	4	1	3	2	1	0	0
G8	0	1	0	1	1	0	1	0
G9	0	1	0	0	0	2	1	0
G10	0	3	0	1	0	0	0	0
G11	0	2	0	1	0	0	0	0
G12	1	1	1	1	2	1	0	0
G13	2	2	0	5	1	0	0	1
G14	1	0	0	0	0	0	0	0
Totals=60	8	16	3	17	7	4	3	2

### Infections With Multiple Strains

Multiple genotypes in one or both genes were detected in at least 14% of samples upon first analysis (rising to 29% when repeat experiments were included), distributed among immunocompetent and immunocompromised individuals. More than one genotype was detected in 11% of European samples, 16% of Gambian samples, 47% of South African samples, and 10% of Hong Kong samples (rising to 24%, 33%, 60%, and 60%, respectively, when repeat experiments are included).

## DISCUSSION

This study focused on the genotype definitions, frequencies, occurrence in mixed infections, geographical distribution and evolution (in terms of linkage disequilibrium and mode of selection) of two hypervariable HCMV genes, UL146 and UL139. Totals of 182 UL146 and 183 UL139 sequences were obtained from a large panel of clinical isolates collected from Africa (South Africa and The Gambia), Asia (Hong Kong), Australia and Europe (various countries). These were used in all analyses, and were supplemented by 168 previously published UL146 and 117 UL139 sequences in analyses of genotype definitions, frequencies and mode of selection.

The UL146 sequences fell into the 14 genotypes described previously [Dolan et al., 2004], and no new genotypes were discovered. Twelve genotypes contained the ELRCXC motif, which has been shown to be essential for receptor binding and IL–8 activity [Clark-Lewis et al., 1991], and 2 contained the NGRCXC motif, which has been shown to be important for interaction with T and B cells [Baggiolini et al., 1997]. The latter genotypes (G5 and G6) are relatively rare, being present in approximately5%of samples. It is not known whether the UL146 genotypes possess different biological properties, and studies to investigate this question are required.

The UL139 sequences grouped into eight genotypes.A recent analysis of 26 clinical samples [Qi et al., 2006] described three major groups (G1, G2, and G3), two of which were divided into subgroups (G1 into G1a, G1b and G1c and G2 into G2a and G2b). Subgroups G1b and G1c in the previous study correspond to G1 in the present study, subgroup G1a corresponds to G4, subgroups G2a and G2b correspond to G6 and G2, respectively, and G3 is named identically in both studies. Thus, apart from the differences in nomenclature, the subgroups [Qi et al., 2006] correlate with a subset of the genotypes in the present study, except that the closely related subgroups G1b and G1c in the former are amalgamated as G1 in the latter. Most of the variation in UL139 is due to substitutions or deletions of variable size near theN terminus. This region is rich in S and T residues that are potentially susceptible to O–glycosylation, and also contains NXS or NXT motifs that are potentially susceptible to N–glycosylation. This suggests that selection may have focused primarily on glycosyl side chains rather than the underlying amino acid sequence. A similar feature characterizes other variable glycoprotein genes, such as UL73 (encoding glycoprotein N(gN)) and UL74 (encoding glycoprotein O(gO)) [Pignatelli et al., 2001, 2003; Mattick et al., 2004].

A region of sequence identity (SETTTGTSSNSS in Fig. 1A) has been noted between the HCMV UL139 protein and CD24, a cellular glycosyl phosphatidylinositol–linked glycoprotein that is involved in B cell activation [Qi et al., 2006]. It is difficult to assess the significance of this observation, especially since 9 of the 12 residues are S or T and the region is not conserved in CD24 orthologues from other mammals. However, the sequence is present in all of the UL139 genotypes identified in the present study, except for G5, and also in the homologous protein from chimpanzee cytomegalovirus (CCMV) (Fig. 1A). Variation in glycosylation has been observed in CD24 and has been linked to differences in cell and tissue specificity [Poncet et al., 1996]. Additional roles for CD24 in apoptosis and cell adhesion have also been suggested, and more recently in regulating the responsiveness of a chemokine receptor, CXCR4 [Schabath et al., 2006; Smith et al., 2006]. The possibility that UL139 may be a CD24 homologue remains intriguing, but, in the absence of functional data, unproven.

Studies of HCMV genotype frequency, including the present one, are usually based on the use of conserved PCR primers, and face limitations as a result. Firstly, there is no guarantee that all genotypes will be detected, since primers are chosen on the basis of alignments of available sequences. Secondly, samples containing more than one strain yield mixed sequences,which when cloned are recovered approximately in proportion to their abundance (although stochastic processes may introduce bias during PCR). Therefore, the absence of a genotype from a particular sample cannot be assured. If anyUL146 or UL139 genotypes have escaped recognition, they may emerge from future studies involving different primers or from whole genome sequencing exercises.

As found in previous studies [reviewed in Puchhammer-Stöckl and Görzer, 2006], mixed infections with different HCMV strains were common. In some samples, a single UL139 genotype and multiple UL146 genotypes, or vice versa, were detected. This could be due to different strains happening to contain the same genotype at one locus but not at the other, or to the limitations of amplifying sequences present as mixtures in unequal proportions. Some samples tested more than once were found to contain additional genotypes not detected in the first experiment, suggesting that the number of mixed infections was underestimated by the methodology used. Mixed infections were more frequently detected from certain regions, namely Hong Kong, South Africa and, to a lesser extent, The Gambia. It is possible that this is a result of higher transmission frequencies. In one study [Beyari et al., 2005], a higher seroprevalence of HCMV in children in Malawi compared to European countries and the USA was taken as possibly reflecting greater opportunities for transmission, although multiple genotypes were detected in only a small number of samples.

The occurrence of mixed infections is being recognized increasingly as potentially significant to the biology of HCMV. This feature adds to the limitations inherent in studies of whether particular genotypes are associated with disease outcome; other features include the number, origin and pathological categorization of samples, the choice of gene, the absence of linkage disequilibrium, and host factors. In light of these limitations, our opinion is that robust evidence in favor of any association between genotype and pathology has proved elusive in the literature. Further work utilizing genotype–specific approaches is required to explore the true frequency of mixed infections, both to validate studies of this type and to determine whether mixed infections have geographical or biological correlates.

Similar to the conclusions drawn from a study on UL73 (encoding gN) [Pignatelli et al., 2003], no statistically significant association of UL146 or UL139 genotypes with geographical origin arose from the analysis. However, this may reflect low sample numbers (albeit much larger than those utilized in previous studies on UL146 and UL139) and the lack of detailed information on ethnic origin. Likewise, investigation of linkage disequilibrium between UL146 and UL139 genotypes was compromised by the small sample number (60) relative to the large number of possible genotype combinations (112). However, no evidence for linkage disequilibrium was obtained, indicating the involvement of recombination in HCMV evolution since the genotypes arose. Taking into account the size of the HCMV gene complement, we agree with the view that very many strains are likely to be circulating in the world [Rasmussen et al., 2003].

The extensive divergence between genotypes and the consequent inability to produce reliable sequence alignments for both UL146 and UL139 in the hypervariable regions compromised assessments of the role of positive selection in generating the genotypes. In contrast, variation within genotypes is low, and identical nucleotide sequences were obtained from geographically distant individuals. The analysis suggests that constraint has been the predominant factor in evolution within genotypes, with positive selection detected only marginally. A previous study [Arav-Boger et al., 2005] involving 30 sequences also concluded that UL146 has evolved under constraint. The sequences of hypervariable genes are stable on short timescales in patients [Hassan-Walker et al., 2004; Stanton et al., 2005] and cell culture [Lurain et al., 2006], consistent with the perception of herpesvirus genomes as relatively slowly evolving [McGeoch et al., 2006]. The most likely scenario for the evolution of HCMV emerging from the literature and from the present study is that the genotypes developed in early human populations (or even earlier), becoming established via founder or bottleneck effects, and have spread, recombined and mixed worldwide in more recent times, with mixed infections being common.
